# Ethyl 4-[3,5-bis­(trifluoro­meth­yl)phen­yl]-6-methyl-2-oxo-1,2,3,4-tetra­hydro­pyrimidine-5-carboxyl­ate

**DOI:** 10.1107/S1600536809019035

**Published:** 2009-05-29

**Authors:** Hoong-Kun Fun, Ching Kheng Quah, M. Babu, B. Kalluraya

**Affiliations:** a X-ray Crystallography Unit, School of Physics, Universiti Sains Malaysia, 11800 USM, Penang, Malaysia; b Department of Studies in Chemistry, Mangalore University, Mangalagangotri, Mangalore 574 199, India

## Abstract

In the title compound, C_16_H_14_F_6_N_2_O_3_, the dihydro­pyrimid­in­one ring adopts an envelope conformation. In the crystal, mol­ecules are linked by N—H⋯O and C—H⋯O hydrogen bonds into a ribbon-like structure along the *b* axis. In the ribbon, a pair of bifurcated acceptor N—H⋯O and C—H⋯O bonds generate an *R*_2_^1^(6) ring motif. Adjacent ribbons are linked *via* C—H⋯F hydrogen bonds.

## Related literature

For general background and the pharmaceutical applications of pyrimidinones, see: Kalluraya & Rai (2003 ▶); Atwal (1990 ▶); Steele *et al.* (1998 ▶); Manjula *et al.* (2004 ▶); Matsuda & Hirao (1965 ▶). For a related structure, see: Fun *et al.* (2009 ▶). For ring conformations, see: Cremer & Pople (1975 ▶). For hydrogen-bond motifs, see: Bernstein *et al.* (1995 ▶). For bond-length data, see: Allen *et al.* (1987 ▶). For the stability of the temperature controller used for the data collection, see: Cosier & Glazer (1986 ▶).
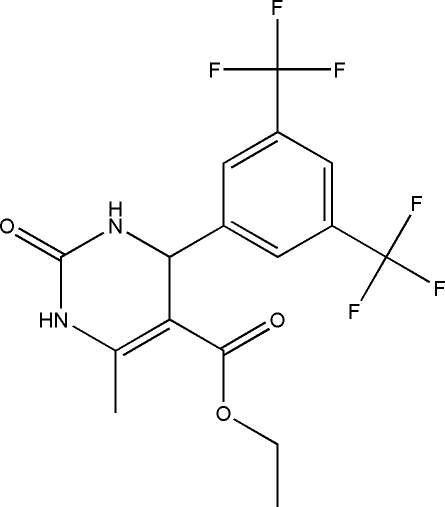


## Experimental

### 

#### Crystal data

C_16_H_14_F_6_N_2_O_3_
*M* *_r_* = 396.29
Monoclinic, 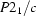

*a* = 12.6876 (2) Å
*b* = 7.3073 (1) Å
*c* = 19.9547 (3) Å
β = 114.443 (1)°
*V* = 1684.23 (5) Å^3^
*Z* = 4
Mo *K*α radiation
μ = 0.15 mm^−1^
*T* = 110 K
0.45 × 0.25 × 0.22 mm

#### Data collection

Bruker SMART APEXII CCD area-detector diffractometer
Absorption correction: multi-scan (**SADABS**; Bruker, 2005 ▶) *T*_min_ = 0.908, *T*_max_ = 0.967
22326 measured reflections
7151 independent reflections
5660 reflections with *I* > 2σ(*I*)
*R*_int_ = 0.025

#### Refinement

*R*[*F* ^2^ > 2σ(*F* ^2^)] = 0.046
*wR*(*F* ^2^) = 0.134
*S* = 1.04
7151 reflections
254 parameters
36 restraints
H atoms treated by a mixture of independent and constrained refinement
Δρ_max_ = 0.49 e Å^−3^
Δρ_min_ = −0.45 e Å^−3^

### 

Data collection: *APEX2* (Bruker, 2005 ▶); cell refinement: *SAINT* (Bruker, 2005 ▶); data reduction: *SAINT*; program(s) used to solve structure: *SHELXTL* (Sheldrick, 2008 ▶); program(s) used to refine structure: *SHELXTL*; molecular graphics: *SHELXTL*; software used to prepare material for publication: *SHELXTL* and *PLATON* (Spek, 2009 ▶).

## Supplementary Material

Crystal structure: contains datablocks global, I. DOI: 10.1107/S1600536809019035/ci2801sup1.cif

Structure factors: contains datablocks I. DOI: 10.1107/S1600536809019035/ci2801Isup2.hkl

Additional supplementary materials:  crystallographic information; 3D view; checkCIF report

## Figures and Tables

**Table 1:** Hydrogen-bond geometry (Å, °)

*D*—H⋯*A*	*D*—H	H⋯*A*	*D*⋯*A*	*D*—H⋯*A*
N1—H1*N*1⋯O1^i^	0.86 (2)	2.05 (2)	2.8641 (13)	157 (1)
N2—H1*N*2⋯O3^ii^	0.86 (2)	2.13 (2)	2.9796 (12)	166 (1)
C5—H5⋯O1^iii^	0.95	2.46	3.3797 (14)	162
C14—H14*B*⋯O3^ii^	0.98	2.46	3.3571 (13)	153
C12—H12*B*⋯F3^iv^	0.99	2.47	3.2308 (15)	133

Symmetry codes: (i) 
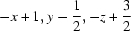
; (ii) 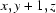; (iii) 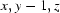; (iv) 
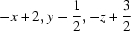
.

**Fig. 1.**
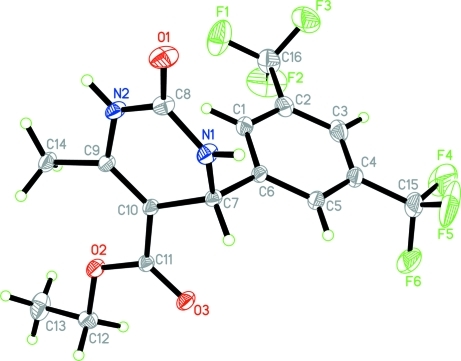
The molecular structure of the title compound, showing 50% probability displacement ellipsoids and the atom-numbering scheme.

**Fig. 2.**
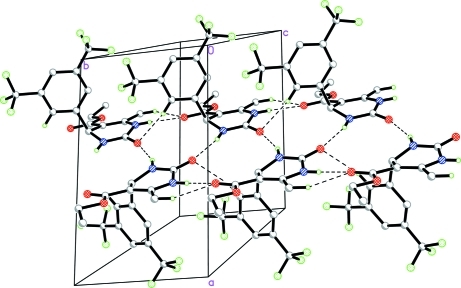
The crystal packing of the title compound, viewed along the [101]. Intermolecular interactions are shown as dashed lines.

**Fig. 3.**
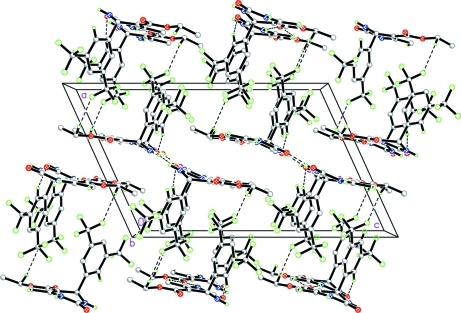
The crystal packing viewed along the b axis. Intermolecular interactions are shown as dashed lines.

## supplementary crystallographic information

### Comment

3,4-Dihydropyrimidinones are compounds that have been drawn wide-spread attention due to their pharmaceutical applications. A variety of dihydropyrimidinone derivatives have been screened for antihypertension (Atwal, 1990) and antibacterial (Matsuda & Hirao, 1965) activities. Michael addition followed by aldol condensation known as the Robinson's annulation is synthetically a very useful reaction for the construction of six-membered cyclic compounds (Kalluraya & Rai, 2003). The common synthetic routes to these compounds generally involve multi-step transformations that are essentially based on the Biginelli condensation methodology (Steele *et al.*, 1998). These pyrimidinones are also associated with activities like calcium channel blocking (Manjula *et al.*, 2004). We report here the crystal structure of the title compound which was synthesized by means of Robinson's annulation employing microwave technique.

The bond lengths (Allen *et al.*, 1987) and angles in the molecule (Fig. 1) are within normal ranges, and are comparable to those observed in a closely related structure (Fun *et al.*, 2009). The dihydropyrimidinone ring adopts an envelope conformation with atom C7 as the flap. The puckering parameters (Cremer & Pople, 1975) are Q = 0.288 (1) Å; Θ = 72.0 (2)° and φ = 52.3 (2)°. The dihedral angle formed by benzene ring (C1—C6) and the N1/C8/N2/C9-/C10 plane is 89.33 (3)°.

In the solid state, the molecules are linked into a ribbon-like structure (Fig. 2) along the [010] by N–H···O and C–H···O hydrogen bonds (Table 1). In the ribbon, C14–H14B···O3 and N2–H1N2···O3 interactions form a pair of bifurcated acceptor bonds, generating an *R*_2_^1^(6) ring motif (Bernstein *et al.*, 1995). The adjacent ribbons are linked via C—H···F hydrogen bonds (Fig. 3).

### Experimental

A mixture of 3,5-bis(trifluoromethyl)benzaldehyde (0.01 mol), ethyl acetoacetate (0.015 mol), thiourea (0.01 mol) and conc. H_2_SO_4_ (2 drops) in absolute alcohol (10 ml) taken in a beaker (100 ml) was zapped inside a MW oven for a duration of 3 minutes (at 160 Watt *i.e*, 25% MW power). The reaction mixture was then allowed to stand at room temperature and the product formed was filtered, washed with ethanol followed by water and dried. Further purification was done by recrystallization from ethanol. Single crystals suitable for X-ray analysis were obtained by slow evaporation of an ethanol solution.

### Refinement

Atoms H1N1 and H1N2 were located in a difference Fourier map and refined freely. The remaining H atoms were positioned geometrically and refined using a riding model, with C-H = 0.95–1.00 Å and *U*_iso_(H) = 1.2 or 1.5*U*_eq_(C). A rotating-group model was applied for the methyl groups. Some of the F atoms show elongated ellipsoids indicating disorder. Attempts to refine a disorder model resulted in large s.u's on occupancy factors and almost the same positional parameters for corresponding F atoms in the major and minor disorder components. Hence the original model was used with the U^ij^ parameters of all F atoms restrained to an approximate isotropic behaviour.

### Crystal data

**Table:**

C_16_H_14_F_6_N_2_O_3_	*F*(000) = 808
*M**_r_* = 396.29	*D*_x_ = 1.563 Mg m^−^^3^
Monoclinic, *P*2_1_/*c*	Mo *K*α radiation, λ = 0.71073 Å
Hall symbol: -P 2ybc	Cell parameters from 9602 reflections
*a* = 12.6876 (2) Å	θ = 3.2–35.0°
*b* = 7.3073 (1) Å	µ = 0.15 mm^−^^1^
*c* = 19.9547 (3) Å	*T* = 110 K
β = 114.443 (1)°	Block, colourless
*V* = 1684.23 (5) Å^3^	0.45 × 0.25 × 0.22 mm
*Z* = 4	

### Data collection

**Table:**

Bruker SMART APEXII CCD area-detector diffractometer	7151 independent reflections
Radiation source: fine-focus sealed tube	5660 reflections with *I* > 2σ(*I*)
graphite	*R*_int_ = 0.025
φ and ω scans	θ_max_ = 35.0°, θ_min_ = 2.2°
Absorption correction: multi-scan (*SADABS*; Bruker, 2005)	*h* = −20→20
*T*_min_ = 0.908, *T*_max_ = 0.967	*k* = −11→11
22326 measured reflections	*l* = −32→32

### Refinement

**Table:**

Refinement on *F*^2^	Primary atom site location: structure-invariant direct methods
Least-squares matrix: full	Secondary atom site location: difference Fourier map
*R*[*F*^2^ > 2σ(*F*^2^)] = 0.046	Hydrogen site location: inferred from neighbouring sites
*wR*(*F*^2^) = 0.134	H atoms treated by a mixture of independent and constrained refinement
*S* = 1.04	*w* = 1/[σ^2^(*F*_o_^2^) + (0.0681*P*)^2^ + 0.4398*P*] where *P* = (*F*_o_^2^ + 2*F*_c_^2^)/3
7151 reflections	(Δ/σ)_max_ = 0.001
254 parameters	Δρ_max_ = 0.49 e Å^−^^3^
36 restraints	Δρ_min_ = −0.45 e Å^−^^3^

### Special details

**Table:**

Experimental. The crystal was placed in the cold stream of an Oxford Cyrosystems Cobra open-flow nitrogen cryostat (Cosier & Glazer, 1986) operating at 110.0 (1) K.
Geometry. All esds (except the esd in the dihedral angle between two l.s. planes) are estimated using the full covariance matrix. The cell esds are taken into account individually in the estimation of esds in distances, angles and torsion angles; correlations between esds in cell parameters are only used when they are defined by crystal symmetry. An approximate (isotropic) treatment of cell esds is used for estimating esds involving l.s. planes.
Refinement. Refinement of F^2^ against ALL reflections. The weighted R-factor wR and goodness of fit S are based on F^2^, conventional R-factors R are based on F, with F set to zero for negative F^2^. The threshold expression of F^2^ > 2sigma(F^2^) is used only for calculating R-factors(gt) etc. and is not relevant to the choice of reflections for refinement. R-factors based on F^2^ are statistically about twice as large as those based on F, and R- factors based on ALL data will be even larger.

### Fractional atomic coordinates and isotropic or equivalent isotropic displacement parameters (Å^2^)

**Table:**

	*x*	*y*	*z*	*U*_iso_*/*U*_eq_	
F1	1.02153 (7)	1.04916 (15)	0.79988 (6)	0.0546 (3)	
F2	1.12550 (8)	0.80663 (18)	0.82674 (7)	0.0625 (3)	
F3	1.08940 (8)	0.94509 (16)	0.90918 (5)	0.0510 (3)	
F4	0.96398 (11)	0.31292 (16)	0.94197 (6)	0.0734 (4)	
F5	0.78290 (13)	0.33990 (15)	0.91770 (7)	0.0721 (4)	
F6	0.83618 (9)	0.18885 (11)	0.84630 (5)	0.0416 (2)	
O1	0.54733 (7)	1.21425 (11)	0.72029 (5)	0.02532 (16)	
O2	0.65993 (7)	0.70237 (10)	0.50892 (4)	0.02120 (15)	
O3	0.63160 (7)	0.49775 (10)	0.58404 (4)	0.01973 (14)	
N1	0.56554 (7)	0.90955 (12)	0.70349 (5)	0.01736 (15)	
H1N1	0.5326 (13)	0.881 (2)	0.7321 (8)	0.024 (4)*	
N2	0.62532 (7)	1.12340 (12)	0.64147 (5)	0.01722 (15)	
H1N2	0.6307 (13)	1.238 (2)	0.6327 (8)	0.025 (4)*	
C1	0.83363 (8)	0.82264 (14)	0.76186 (5)	0.01796 (16)	
H1	0.8281	0.9301	0.7338	0.022*	
C2	0.93726 (9)	0.77888 (16)	0.82033 (5)	0.02191 (19)	
C3	0.94750 (10)	0.62163 (17)	0.86221 (6)	0.0247 (2)	
H3	1.0185	0.5927	0.9024	0.030*	
C4	0.85233 (10)	0.50878 (15)	0.84400 (5)	0.02311 (19)	
C5	0.74728 (9)	0.55033 (14)	0.78490 (5)	0.01934 (17)	
H5	0.6829	0.4704	0.7727	0.023*	
C6	0.73721 (8)	0.70860 (13)	0.74415 (5)	0.01511 (15)	
C7	0.62140 (7)	0.75947 (13)	0.68172 (5)	0.01453 (15)	
H7	0.5695	0.6501	0.6705	0.017*	
C8	0.57787 (8)	1.08737 (14)	0.69151 (5)	0.01731 (16)	
C9	0.64143 (7)	0.99139 (13)	0.59672 (5)	0.01476 (15)	
C10	0.63470 (7)	0.81180 (12)	0.61226 (5)	0.01376 (14)	
C11	0.64206 (7)	0.65599 (13)	0.56809 (5)	0.01450 (15)	
C12	0.66444 (9)	0.55645 (15)	0.46087 (5)	0.02057 (18)	
H12A	0.5860	0.5072	0.4317	0.025*	
H12B	0.7146	0.4556	0.4900	0.025*	
C13	0.71342 (13)	0.64003 (19)	0.41112 (7)	0.0337 (3)	
H13A	0.7119	0.5496	0.3745	0.051*	
H13B	0.7935	0.6785	0.4404	0.051*	
H13C	0.6669	0.7466	0.3861	0.051*	
C14	0.66536 (10)	1.06995 (14)	0.53478 (5)	0.02129 (18)	
H14A	0.7385	1.0205	0.5369	0.032*	
H14B	0.6709	1.2035	0.5394	0.032*	
H14C	0.6023	1.0371	0.4876	0.032*	
C15	0.85946 (13)	0.33881 (18)	0.88774 (7)	0.0338 (3)	
C16	1.04253 (10)	0.8954 (2)	0.83865 (7)	0.0314 (2)	

### Atomic displacement parameters (Å^2^)

**Table:**

	*U*^11^	*U*^22^	*U*^33^	*U*^12^	*U*^13^	*U*^23^
F1	0.0302 (4)	0.0609 (6)	0.0583 (6)	−0.0195 (4)	0.0037 (4)	0.0275 (5)
F2	0.0249 (4)	0.0770 (8)	0.0886 (8)	−0.0072 (4)	0.0266 (5)	−0.0217 (7)
F3	0.0464 (5)	0.0600 (7)	0.0342 (4)	−0.0237 (5)	0.0044 (4)	−0.0086 (4)
F4	0.0821 (8)	0.0439 (6)	0.0514 (6)	−0.0039 (5)	−0.0152 (5)	0.0284 (5)
F5	0.1372 (11)	0.0420 (6)	0.0831 (8)	0.0306 (6)	0.0916 (9)	0.0335 (6)
F6	0.0642 (6)	0.0183 (4)	0.0462 (5)	0.0014 (4)	0.0267 (4)	0.0044 (3)
O1	0.0308 (4)	0.0193 (4)	0.0354 (4)	0.0011 (3)	0.0233 (3)	−0.0065 (3)
O2	0.0373 (4)	0.0144 (3)	0.0191 (3)	−0.0018 (3)	0.0188 (3)	−0.0016 (3)
O3	0.0293 (3)	0.0120 (3)	0.0219 (3)	−0.0003 (3)	0.0147 (3)	0.0004 (2)
N1	0.0207 (3)	0.0156 (4)	0.0226 (3)	−0.0005 (3)	0.0157 (3)	−0.0004 (3)
N2	0.0235 (4)	0.0112 (3)	0.0226 (3)	−0.0007 (3)	0.0151 (3)	−0.0012 (3)
C1	0.0194 (4)	0.0180 (4)	0.0178 (3)	−0.0003 (3)	0.0091 (3)	0.0019 (3)
C2	0.0198 (4)	0.0257 (5)	0.0193 (4)	0.0002 (4)	0.0072 (3)	0.0008 (4)
C3	0.0265 (5)	0.0266 (5)	0.0185 (4)	0.0059 (4)	0.0069 (3)	0.0033 (4)
C4	0.0358 (5)	0.0166 (4)	0.0181 (4)	0.0036 (4)	0.0123 (4)	0.0029 (3)
C5	0.0284 (4)	0.0149 (4)	0.0176 (4)	−0.0010 (3)	0.0123 (3)	0.0005 (3)
C6	0.0203 (4)	0.0134 (4)	0.0146 (3)	0.0000 (3)	0.0103 (3)	0.0000 (3)
C7	0.0178 (3)	0.0128 (4)	0.0164 (3)	−0.0020 (3)	0.0104 (3)	−0.0009 (3)
C8	0.0175 (4)	0.0165 (4)	0.0218 (4)	−0.0004 (3)	0.0120 (3)	−0.0025 (3)
C9	0.0175 (3)	0.0126 (4)	0.0161 (3)	−0.0003 (3)	0.0088 (3)	−0.0001 (3)
C10	0.0174 (3)	0.0120 (4)	0.0143 (3)	−0.0008 (3)	0.0090 (3)	0.0000 (3)
C11	0.0167 (3)	0.0138 (4)	0.0143 (3)	−0.0002 (3)	0.0077 (3)	0.0001 (3)
C12	0.0268 (4)	0.0195 (4)	0.0191 (4)	−0.0007 (4)	0.0131 (3)	−0.0049 (3)
C13	0.0535 (7)	0.0306 (6)	0.0308 (5)	0.0104 (5)	0.0313 (5)	0.0070 (5)
C14	0.0348 (5)	0.0140 (4)	0.0210 (4)	−0.0005 (4)	0.0175 (4)	0.0021 (3)
C15	0.0523 (7)	0.0227 (6)	0.0262 (5)	0.0061 (5)	0.0159 (5)	0.0083 (4)
C16	0.0201 (4)	0.0404 (7)	0.0290 (5)	−0.0035 (4)	0.0054 (4)	0.0029 (5)

### Geometric parameters (Å, °)

**Table:**

F1—C16	1.3271 (17)	C3—C4	1.3809 (17)
F2—C16	1.3388 (16)	C3—H3	0.95
F3—C16	1.3317 (15)	C4—C5	1.3994 (15)
F4—C15	1.3334 (17)	C4—C15	1.4991 (16)
F5—C15	1.3348 (17)	C5—C6	1.3890 (13)
F6—C15	1.3302 (16)	C5—H5	0.95
O1—C8	1.2344 (11)	C6—C7	1.5278 (13)
O2—C11	1.3350 (10)	C7—C10	1.5129 (11)
O2—C12	1.4508 (12)	C7—H7	1.00
O3—C11	1.2210 (12)	C9—C10	1.3593 (13)
N1—C8	1.3420 (13)	C9—C14	1.5034 (12)
N1—C7	1.4659 (12)	C10—C11	1.4663 (12)
N1—H1N1	0.860 (15)	C12—C13	1.5017 (15)
N2—C9	1.3859 (12)	C12—H12A	0.99
N2—C8	1.3879 (11)	C12—H12B	0.99
N2—H1N2	0.866 (17)	C13—H13A	0.98
C1—C2	1.3867 (14)	C13—H13B	0.98
C1—C6	1.3993 (13)	C13—H13C	0.98
C1—H1	0.95	C14—H14A	0.98
C2—C3	1.3949 (16)	C14—H14B	0.98
C2—C16	1.4961 (16)	C14—H14C	0.98
			
C11—O2—C12	117.75 (8)	C9—C10—C11	125.94 (8)
C8—N1—C7	124.42 (7)	C9—C10—C7	119.63 (8)
C8—N1—H1N1	118.3 (11)	C11—C10—C7	114.42 (8)
C7—N1—H1N1	116.5 (11)	O3—C11—O2	123.19 (8)
C9—N2—C8	123.71 (8)	O3—C11—C10	122.54 (8)
C9—N2—H1N2	120.0 (10)	O2—C11—C10	114.26 (8)
C8—N2—H1N2	115.0 (10)	O2—C12—C13	106.15 (9)
C2—C1—C6	120.04 (9)	O2—C12—H12A	110.5
C2—C1—H1	120.0	C13—C12—H12A	110.5
C6—C1—H1	120.0	O2—C12—H12B	110.5
C1—C2—C3	120.99 (10)	C13—C12—H12B	110.5
C1—C2—C16	120.94 (10)	H12A—C12—H12B	108.7
C3—C2—C16	118.04 (10)	C12—C13—H13A	109.5
C4—C3—C2	118.67 (9)	C12—C13—H13B	109.5
C4—C3—H3	120.7	H13A—C13—H13B	109.5
C2—C3—H3	120.7	C12—C13—H13C	109.5
C3—C4—C5	121.09 (10)	H13A—C13—H13C	109.5
C3—C4—C15	120.40 (10)	H13B—C13—H13C	109.5
C5—C4—C15	118.51 (11)	C9—C14—H14A	109.5
C6—C5—C4	119.92 (9)	C9—C14—H14B	109.5
C6—C5—H5	120.0	H14A—C14—H14B	109.5
C4—C5—H5	120.0	C9—C14—H14C	109.5
C5—C6—C1	119.28 (9)	H14A—C14—H14C	109.5
C5—C6—C7	120.29 (8)	H14B—C14—H14C	109.5
C1—C6—C7	120.42 (8)	F6—C15—F4	106.15 (12)
N1—C7—C10	109.47 (7)	F6—C15—F5	105.53 (12)
N1—C7—C6	111.10 (7)	F4—C15—F5	107.51 (12)
C10—C7—C6	111.98 (7)	F6—C15—C4	112.10 (10)
N1—C7—H7	108.0	F4—C15—C4	112.97 (12)
C10—C7—H7	108.0	F5—C15—C4	112.09 (11)
C6—C7—H7	108.0	F1—C16—F3	106.23 (12)
O1—C8—N1	124.21 (8)	F1—C16—F2	106.83 (12)
O1—C8—N2	120.35 (9)	F3—C16—F2	106.23 (11)
N1—C8—N2	115.41 (8)	F1—C16—C2	113.32 (10)
C10—C9—N2	119.02 (8)	F3—C16—C2	112.12 (10)
C10—C9—C14	127.54 (8)	F2—C16—C2	111.64 (12)
N2—C9—C14	113.44 (8)		
			
C6—C1—C2—C3	0.11 (15)	N2—C9—C10—C7	−5.85 (13)
C6—C1—C2—C16	177.92 (10)	C14—C9—C10—C7	173.99 (9)
C1—C2—C3—C4	0.49 (16)	N1—C7—C10—C9	26.13 (11)
C16—C2—C3—C4	−177.37 (10)	C6—C7—C10—C9	−97.55 (10)
C2—C3—C4—C5	−0.12 (15)	N1—C7—C10—C11	−155.34 (7)
C2—C3—C4—C15	−179.77 (10)	C6—C7—C10—C11	80.98 (10)
C3—C4—C5—C6	−0.86 (15)	C12—O2—C11—O3	1.37 (14)
C15—C4—C5—C6	178.79 (9)	C12—O2—C11—C10	−177.99 (8)
C4—C5—C6—C1	1.46 (13)	C9—C10—C11—O3	−177.59 (9)
C4—C5—C6—C7	−177.28 (8)	C7—C10—C11—O3	4.00 (13)
C2—C1—C6—C5	−1.09 (14)	C9—C10—C11—O2	1.78 (13)
C2—C1—C6—C7	177.64 (8)	C7—C10—C11—O2	−176.63 (8)
C8—N1—C7—C10	−31.69 (12)	C11—O2—C12—C13	−166.51 (9)
C8—N1—C7—C6	92.51 (11)	C3—C4—C15—F6	−120.88 (13)
C5—C6—C7—N1	104.84 (9)	C5—C4—C15—F6	59.46 (15)
C1—C6—C7—N1	−73.88 (10)	C3—C4—C15—F4	−1.01 (17)
C5—C6—C7—C10	−132.40 (9)	C5—C4—C15—F4	179.34 (11)
C1—C6—C7—C10	48.88 (11)	C3—C4—C15—F5	120.64 (14)
C7—N1—C8—O1	−167.27 (9)	C5—C4—C15—F5	−59.01 (15)
C7—N1—C8—N2	14.68 (14)	C1—C2—C16—F1	7.46 (17)
C9—N2—C8—O1	−167.38 (9)	C3—C2—C16—F1	−174.67 (11)
C9—N2—C8—N1	10.76 (14)	C1—C2—C16—F3	127.71 (12)
C8—N2—C9—C10	−14.76 (14)	C3—C2—C16—F3	−54.42 (16)
C8—N2—C9—C14	165.38 (9)	C1—C2—C16—F2	−113.22 (13)
N2—C9—C10—C11	175.81 (8)	C3—C2—C16—F2	64.65 (14)
C14—C9—C10—C11	−4.35 (15)		

### Hydrogen-bond geometry (Å, °)

**Table:**

*D*—H···*A*	*D*—H	H···*A*	*D*···*A*	*D*—H···*A*
N1—H1N1···O1^i^	0.86 (2)	2.05 (2)	2.8641 (13)	157 (1)
N2—H1N2···O3^ii^	0.86 (2)	2.13 (2)	2.9796 (12)	166 (1)
C5—H5···O1^iii^	0.95	2.46	3.3797 (14)	162
C14—H14B···O3^ii^	0.98	2.46	3.3571 (13)	153
C12—H12B···F3^iv^	0.99	2.47	3.2308 (15)	133

Symmetry codes: (i) −*x*+1, *y*−1/2, −*z*+3/2; (ii) *x*, *y*+1, *z*; (iii) *x*, *y*−1, *z*; (iv) −*x*+2, *y*−1/2, −*z*+3/2.
